# mHealth intervention to improve the continuum of maternal and perinatal care in rural Guatemala: a pragmatic, randomized controlled feasibility trial

**DOI:** 10.1186/s12978-018-0554-z

**Published:** 2018-07-04

**Authors:** Boris Martinez, Enma Coyote Ixen, Rachel Hall-Clifford, Michel Juarez, Ann C. Miller, Aaron Francis, Camilo E. Valderrama, Lisa Stroux, Gari D. Clifford, Peter Rohloff

**Affiliations:** 1 Wuqu’ Kawoq | Maya Health Alliance, 2a. Calle 5-43 Zona 1, Santiago Sacatepéquez, Guatemala; 2 Departments of Sociology and Anthropology and Public Health, Agnes Scott College, Decatur, GA USA; 3 Department of Global Health and Social Medicine, Harvard Medical School, Boston, MA USA; 4 Department of Biomedical Informatics, Emory University, Atlanta, GA USA; 5 Institute of Biomedical Engineering, Department of Engineering Science, University of Oxford, Oxford, UK; 6 Department of Biomedical Engineering, Georgia Institute of Technology and Emory University, Atlanta, GA USA; 7 Division of Global Health Equity, Brigham and Women’s Hospital, Boston, MA USA; 8 Department of Medicine, Saint Peter’s University Hospital, New Brunswick, NJ USA

**Keywords:** Barriers to care, Lay midwives, mHealth, Perinatal monitoring, Referral system, Guatemala, Indigenous, Resource-constrained healthcare, Traditional birth attendants

## Abstract

**Background/objective:**

Guatemala’s indigenous Maya population has one of the highest perinatal and maternal mortality rates in Latin America. In this population most births are delivered at home by traditional birth attendants (TBAs), who have limited support and linkages to public hospitals. The goal of this study was to characterize the detection of maternal and perinatal complications and rates of facility-level referral by TBAs, and to evaluate the impact of a mHealth decision support system on these rates.

**Methods:**

A pragmatic one-year feasibility trial of an mHealth decisions support system was conducted in rural Maya communities in collaboration with TBAs. TBAs were individually randomized in an unblinded fashion to either early-access or later-access to the mHealth system. TBAs in the early-access arm used the mHealth system throughout the study. TBAs in the later-access arm provided usual care until crossing over uni-directionally to the mHealth system at the study midpoint. The primary study outcome was the monthly rate of referral to facility-level care, adjusted for birth volume.

**Results:**

Forty-four TBAs were randomized, 23 to the early-access arm and 21 to the later-access arm. Outcomes were analyzed for 799 pregnancies (early-access 425, later-access 374). Monthly referral rates to facility-level care were significantly higher among the early-access arm (median 33 referrals per 100 births, IQR 22–58) compared to the later-access arm (median 20 per 100, IQR 0–30) (*p* = 0.03). At the study midpoint, the later-access arm began using the mHealth platform and its referral rates increased (median 34 referrals per 100 births, IQR 5–50) with no significant difference from the early-access arm (*p* = 0.58). Rates of complications were similar in both arms, except for hypertensive disorders of pregnancy, which were significantly higher among TBAs in the early-access arm (RR 3.3, 95% CI 1.10–9.86).

**Conclusions:**

Referral rates were higher when TBAs had access to the mHealth platform. The introduction of mHealth supportive technologies for TBAs is feasible and can improve detection of complications and timely referral to facility-care within challenging healthcare delivery contexts.

**Trial registration:**

Clinicaltrials.gov NCT02348840.

## Plain English Summary

The indigenous Maya population in Guatemala has one of the highest rates of dying from complications during pregnancy and childbirth in Latin America. Due to many contributing factors, most Maya women opt for home deliveries attended by traditional birth attendants (TBAs). TBAs are required to arrange referrals when complications arise, however they usually lack support from and linkages to public hospitals. We have designed a smartphone application that provides support and guidance to TBAs while they evaluate patients. In this study, we evaluated the impact of this smartphone application on utilization of higher-level medical care for rural patients in Guatemala.

Collaborating TBAs were randomly assigned to either have early-access or later-access to the smartphone application. TBAs in the early-access group used the application for the whole study period. TBAs in the later-access group initially provided standard care to patients and then, half-way through the study, they began using the smartphone application. An emergency on-call team was available for TBAs in both groups to provide support during the referral process.

We found that referral rates for pregnancy and childbirth complications were higher when TBAs had access to the smartphone application. Our findings show that the introduction of such technology in the practice of TBAs is feasible and can lead to improvements in the detection of pregnancy and childbirth complications and timely referral of patients to hospital care.

## Background

Improving maternal and neonatal health in resource-limited settings is a key priority in global health [1]. Around 300,000 women die each year from pregnancy or labor-related complications, most of these in Low- and Middle-Income Countries (LMICs) [2]. Perinatal deaths (stillbirths and early neonatal death) are similarly concentrated in LMICs, at an overall rate of 5.4 million per year [3]. Guatemala, the most populous country in Central America, has one of the highest perinatal and maternal mortality rates in Latin America [4, 5]. These poor outcomes particularly impact Guatemala’s rural indigenous Maya population, where both perinatal and maternal mortality rates are markedly higher than for Guatemala’s non-indigenous population [6, 7].

Contributing factors to these disparities include lack of rural infrastructure for early detection and referral of maternal and neonatal complications and systemic discrimination against Maya women in healthcare facilities, leading to a strong cultural preference for home births [8–10]. Indeed, at least 50% of births in rural Maya communities in Guatemala occur in the home under the care of Traditional Birth Attendants (TBAs) [7, 11].

Therefore, addressing the continuum of maternal and perinatal care in Guatemala requires not only increasing access to facility-level births, but also improving the early detection of complications in community-based care and the formal linkages of TBAs to higher levels of care [9–12]. A few studies have demonstrated the promise of mHealth systems for improving maternal and neonatal outcomes in LMICs [13]. However, with a few exceptions, most of these have focused on community health workers with a higher level of literacy and formal healthcare system engagement than is typical for TBAs [14, 15].

We have recently designed a low-cost, real-time decision support smartphone application for use by non-literate Maya TBAs, which is augmented by input from commercially-available sensors, including 1-D Doppler ultrasound, pulse oximetry, and oscillometric blood pressure [16, 17]. In this study, we report results from a pragmatic feasibility trial of this system by 44 TBAs in rural Guatemala. The goals of the trial were to characterize, for the first time, baseline rates of complication detection and facility-level referral by TBAs in rural Guatemala, and to evaluate the impact of the mHealth system on these rates. We hypothesized that the system would improve maternal and perinatal complication detection and referral rates to facility-level care.

## Methods

### Study design

This was a pragmatic, randomized controlled trial assessing the effectiveness of a mHealth decision support system to improve maternal and perinatal complication detection and referral rates to facility-level care by TBAs. Forty-four indigenous Maya TBAs from one rural municipality (Tecpán, Chimaltenango, pop. 95,000) in rural Guatemala participated. TBAs were randomized to have either early- or later-access to the mHealth platform. TBAs in the early-access arm used the mHealth platform through the whole study duration. Initially, TBAs in the later access-arm provided usual care and subsequently transitioned to use of the mHealth platform in a unidirectional cross-over. The study was conducted in collaboration with Maya Health Alliance (MHA), a Guatemalan primary health care organization with a clinical center in Tecpán. The study was approved by the Institutional Review Boards of MHA (Protocol number WK-2015-001) and Emory University (Protocol number IRB00076231). The study protocol and replication data set are available online at: https://doi.org/10.7910/DVN/VCJKH7. The trial was registered at ClinicalTrials.gov, NCT02348840.

### Participants

All TBAs aged 18–65 years who were independently practicing in the Tecpán municipality, who had attended at least 5 deliveries per year in the previous 5 years, and who held a valid license to practice issued by local health authorities were eligible to participate in the study. A list of 150 TBAs from the study area was produced in collaboration with local health officials. Eighty-six TBAs did not met eligibility criteria. All TBAs meeting eligibility criteria (*n* = 64) were invited to attend an informational session led by research staff, 49 TBAs attended this meeting. Subsequently, 44 of them (29% of total TBAs, 69% of eligible TBAs) agreed to participate, and written informed consent was obtained by study staff bilingual in Spanish and Kaqchikel Maya, the language spoken by most of the TBAs.

During the study, all TBAs continued to provide usual home-based care to their patients. TBAs alerted study staff to new pregnant patients in their practice. A study staff member bilingual in Kaqchikel and Spanish subsequently visited patients (and the biological father, when available) in the home to explain the study and obtained written informed consent for collection of individual-level health information and pregnancy perinatal outcomes data. All pregnant patients over 18 years of age and under the care of a TBA were eligible to participate.

### Randomization and masking

Stratified randomization of TBAs to two study arms (early-access or later-access to the mHealth technology) was performed using a computer-based randomization process. A Montecarlo statistical analysis using a rank sum test was used to ensure median ages, community origin, and distance to municipality were not significantly different between study arms. The allocation and assignment procedures were performed by a study author (GC) prior to meeting participants and not otherwise involved in the recruitment of participants or daily conduct of field work. Due to the pragmatic nature of a trial involving access to mHealth technology, neither TBAs, pregnant subjects, nor study personnel could be blinded to allocation. However, many TBAs resided/practiced in a single isolated rural settlement within the municipal catchment area, with minimal overlap with other TBAs. In addition, when more than one TBA resided/practiced within the same settlement, they were allocated to the same study arm. This resulted in 23 TBAs from 13 villages being assigned to the early-access arm, and 21 TBAs from 15 mutually exclusive villages being assigned to the later-access arm.

### Intervention

**Fig. 1:**
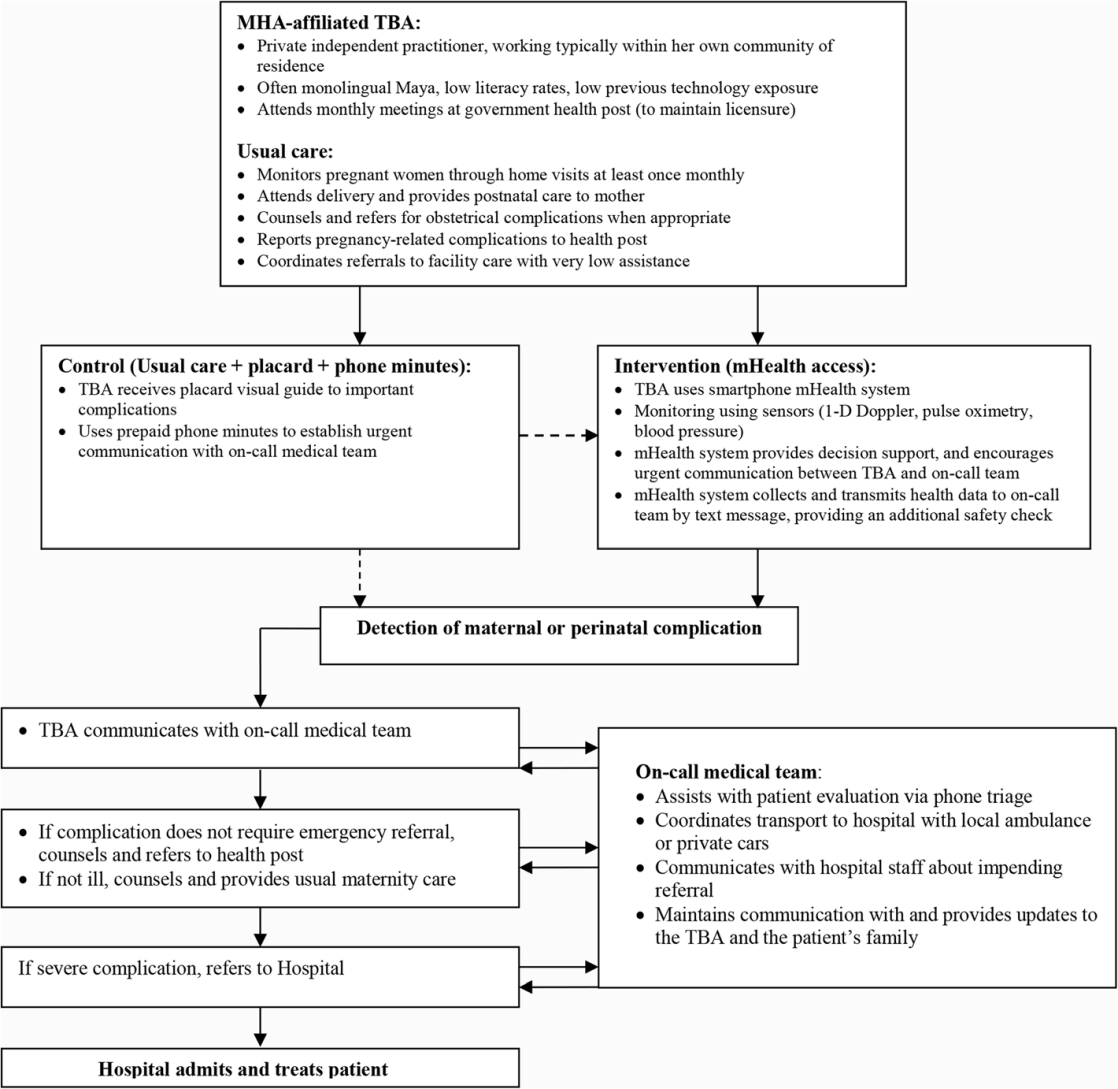
A schematic view of the traditional birth attendant obstetric referral strategy. Figure outlines the emergency referral workflow between TBAs and supporting medical team and details the integration of the mHealth system into this workflow

A perinatal monitoring mHealth platform was introduced in the daily practice of participating TBAs in a unidirectional cross-over study design. We hypothesized that such a system would improve timely and accurate TBA-initiated referrals to higher levels of care, measured as referral rates per number of births and as the proportion of successful referrals respectively, by improving the diagnostic capabilities of participating TBAs and connecting them with an existing referral network. Use of the mHealth platform by TBAs was supported by an existing referral support structure at MHA outlined in Fig. 1, consisting of an on-call clinical team who provided triage support and coordinated transportation to hospital if referral was needed. This wrap-around support structure at MHA existed prior to the mHealth intervention, helping with emergency care for MHA patients and patients referred from collaborating health districts. However, prior to this study, attempts had not been made to integrate TBAs into this network.

The mHealth platform used in this study was developed for the Android operating system (4.2.2; Google Inc.) and installed on Samsung S3 mini smartphone devices (costing about $100). This smartphone platform also provided integrated use of peripheral sensor devices, including a pulse oximeter (Onyx II, Model 9560, Nonin Medical, Inc.), a hand-held 1-dimensional Doppler ultrasound device (AngelSounds Fetal Doppler JPD-100 s, Jumper Medical Co., Ltd.), and—via a customized camera application—a self-inflating oscillometric blood pressure cuff (Omron M7, OMRON Healthcare). We have previously described in more detail the technical aspects of the design and end-user testing by TBAs for this platform [16, 17].

**Fig. 2:**
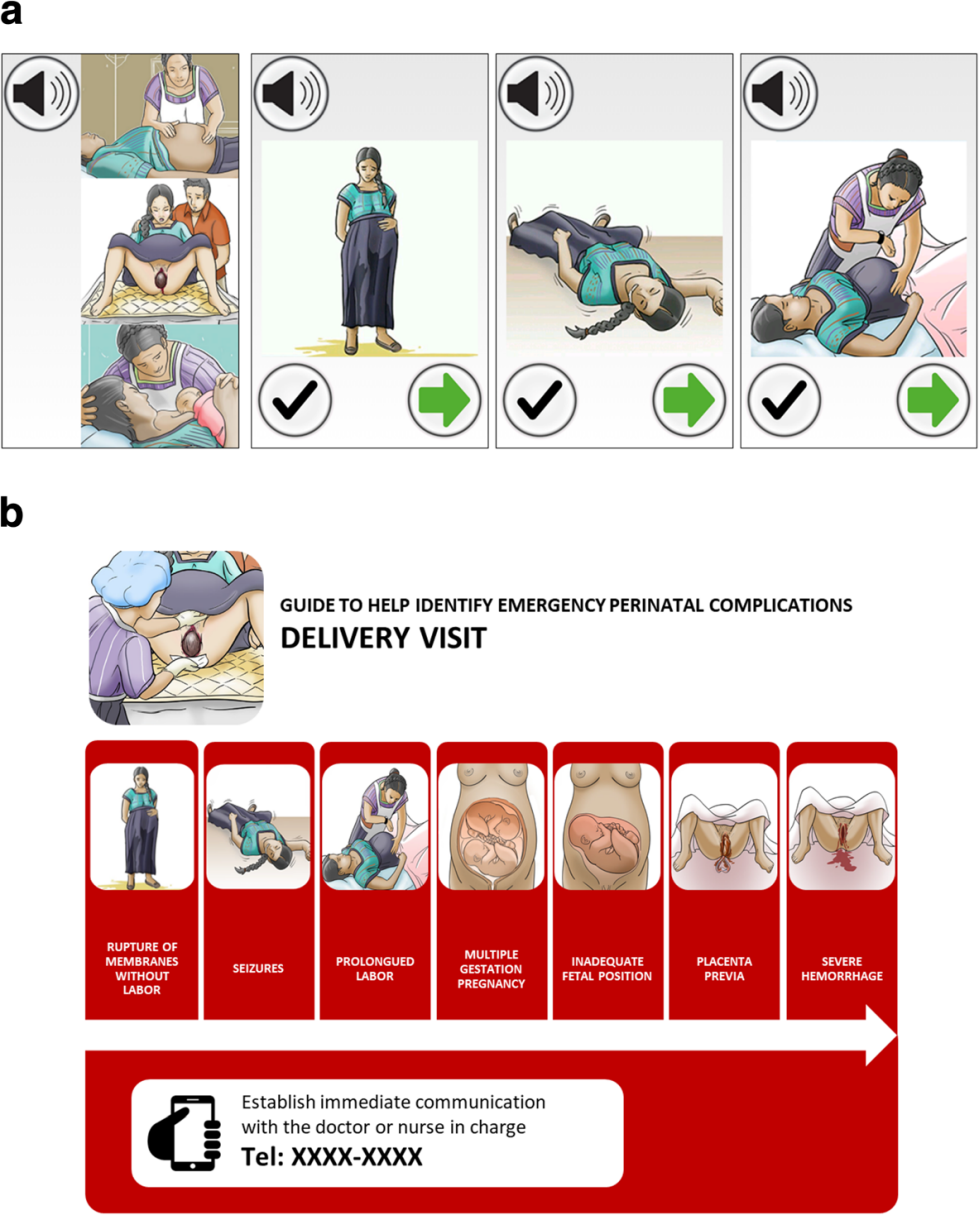
Example of decision support workflows for facilitating obstetric emergency referral. **a** Shows an example of workflow on the mHealth platform. In the left-most illustration, the TBA selects the appropriate visit type (prenatal, perinatal, postnatal). The TBA is then guided through a series of pictorial warning signs, with audio prompts as necessary. Checking a warning sign generates an emergency call and text-message to on-call team. **b** Shows the same pictorial partial workflow through warning signs for a typical perinatal visit for TBAs using the visit placard in the later-access arm (during the non-intervention period), where an emergency telephone number is provided. All images were adapted from existing public sector resources in Guatemala [33]

In terms of decision support, the smartphone application allowed collection of simple demographics, maternal and perinatal symptoms and clinical signs, maternal vital signs (pulse, oxygen saturation, systolic and diastolic blood pressure), and the fetal heart rate. While using the application, TBAs are guided through a pictographic list of common maternal and perinatal complications grouped by visit type, as outlined in Table 1 and depicted in Fig. 2a. Checking complications triggered automatic communications with the on-call clinical team by voice call or text message, as outlined in Table 1. Vital sign abnormalities also triggered alert text messages to the on-call team (maternal: heart rate ≤ 60 or ≥ 100 bpm, oxygen saturation ≤ 90%, systolic blood pressure ≤ 70 or ≥ 140 mmHg, diastolic blood pressure ≥ 90 mmHg; fetal heart rate ≤ 120 or ≥ 160 bpm).

**Table 1:** Guided list of complications included in the mHealth platform by visit type

Prenatal	Perinatal	Postnatal
Seizures^a^ Hemorrhage^a^ Premature rupture of membranes^a^ Difficulty breathing^a^ Abdominal pain Blurred vision Fever Edema Severe headache	Seizures^a^ Rupture of membranes without labor^a^ Prolonged labor^a^ Multiple pregnancy^a^ Fetal malpresentation or malposition^a^ Placenta previa^a^ Nuchal cord^a^ Hemorrhage^a^ Placental retention^a^ Previous C-section^a^ Difficulty breathing^a^ Severe abdominal pain^a^ Blurred vision^a^ Fever^a^ Edema^a^ Severe headache^a^	Maternal: Seizures^a^ Hemorrhage^a^ Difficulty breathing^a^ Abdominal pain Fever Blurred vision Headache Neonatal: Difficulty breathing^a^ Fever^a^ Hypothermia^a^ Low-birth weight^a^ Very small baby^a^ Jaundice Breastfeeding difficulties Umbilical infection

Complications marked with an superscripted (^a^) triggered an automatic emergency call to the on-call clinical team (interrupting the work-flow of the visit). Remaining complications triggered an alert screen upon conclusion of the visit requesting a call to the team. All complications data were also automatically conveyed to the on-call team by a text message at the end of the visit encounter. Adapted from Stroux L et al [16]

TBAs randomized to the early-access arm (*n* = 23) participated in a four-day training led by study nurses, where they first refreshed key medical concepts related to perinatal complications, including risk factors for maternal and neonatal morbidity and mortality, indications for referral to higher levels of medical care, importance of timely assessment, and use of the smartphone decision support platform (Fig. 2a, Table 1). This and subsequent training described below were similar in design to other courses previously conducted by MHA, relying on adult-learning and low-literacy strategies such as interactive narrative stories and pictorial informational and instructional material. After completion of this study training, three standardized patient encounters were used to evaluate each participant’s use of the mHealth platform when facing common maternal and perinatal complications. Each standardized encounter was observed by a study staff member, using an observation checklist with 60 discrete tasks (minimum passing score of 90%), who assessed the TBA’s ability to detect the complication, use the mHealth platform, and establish communication with the emergency on-call team. Six of 23 TBAs (26%) failed the first evaluation, received a two-day retraining session, and subsequently passed.

**Fig. 3:**
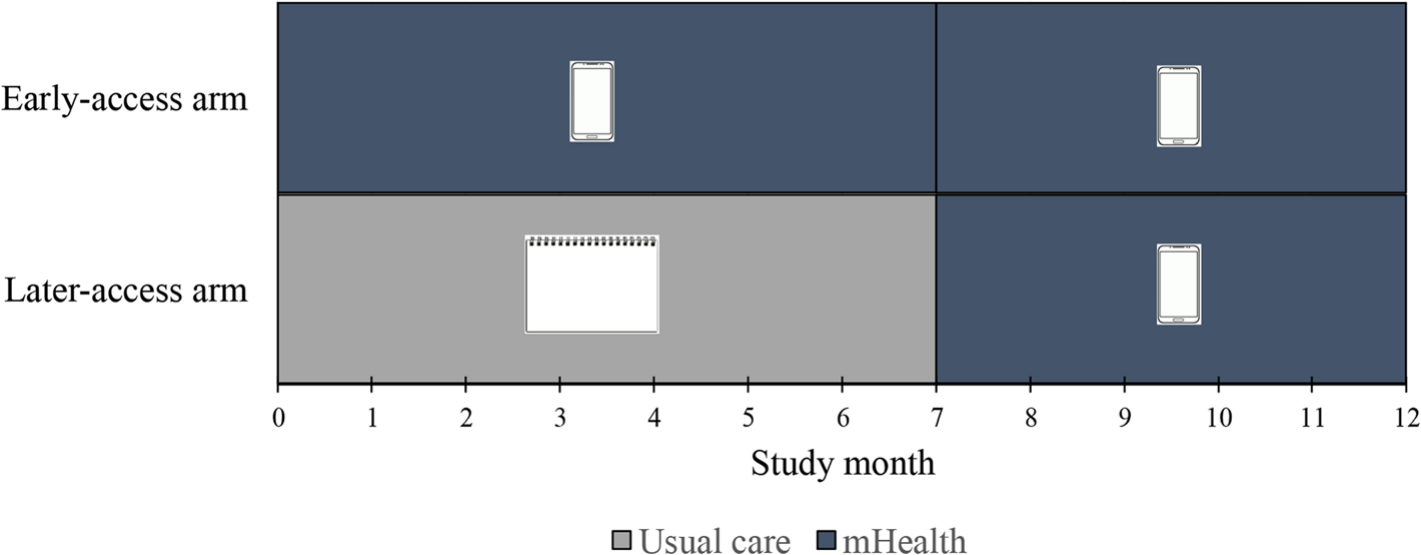
Intervention design. TBAs in the early-access arm used the mHealth platform (intervention) through the whole study duration, depicted as a cell phone device in this and related figures. TBAs in the later-access arm provided usual care during the first 7 months of the study (non-intervention period), assisted with a placard-based complication guide (depicted as a notebook sheet in this and related figures), and subsequently crossed into use of the mHealth platform (intervention period)

TBAs randomized to the later-access arm (*n* = 21) received a separate, two-day training containing the same medical content as the early-access arm, less the mHealth content. Instead of training on the mHealth system, they received a laminated placard containing the same images of the maternal and perinatal complications used in the smartphone system, as well as emergency contact numbers and instructions for initiating referrals (Fig. 2b). Similar standardized patient encounters and an evaluation checklist were also used to evaluate each participant’s use of the placard-based complication guide. All TBAs passed the evaluation and subsequently received a copy of the placard. After month 7 of the study, TBAs in the later-access arm received training on the smartphone platform, as described above, and began to use the mHealth platform (Fig. 3). Two of 21 TBAs (10%) failed the first evaluation, but were subsequently retrained and passed.

### Outcomes

The primary outcome was the number of monthly referrals to facility-level care from TBAs for maternal and perinatal complications, adjusted by the monthly birth volume. A secondary outcome was the proportion of referrals which were completed, defined as successfully receiving facility-based care after a TBA-initiated referral. A computer-based list of births attended was updated every two weeks by the study team with direct input from each TBA. Referrals outcomes were adjudicated by the study team with input from the TBA, and were considered successful if the subject received facility-level medical care. Outcomes data collection was overseen by study staff (physician and nurse), and monthly monitored for missing data, correct data entry errors, and to ensure consistency in collection and reporting over time.

The mHealth intervention involved the use of standard smartphones and CE-approved peripheral sensors (1-D Doppler ultrasound, pulse oximeter, and oscillometric blood pressure cuff). Therefore, no adverse study-related outcomes were anticipated. Nevertheless, study staff maintained a spreadsheet of all clinical outcomes from referrals, as well as morbidity and mortality data reported by TBAs, updated daily. A study physician not otherwise involved in the intervention reviewed these data, determining whether an adverse clinical outcome was possibly study-related. None of the reported adverse events were adjudicated as study related.

### Statistical analysis

This was an exploratory, pragmatic pilot trial designed to explore baseline maternal and perinatal referral rates by practicing TBAs in rural Guatemala to facility-level care, and to explore the feasibility of and impact from use of a smartphone decision support application by these TBAs. As such, no formal sample size calculations were conducted [18]. Rather, the study was conducted within a single municipal catchment area, defined as a high-priority target by the implementing partner given locally high rates of maternal and perinatal complications, and could be used to inform future sample size calculations. Within this target area, initial estimates from the partner were that 50 practicing TBAs would meet eligibility criteria and, with an average of 10 births per year per TBA, we anticipated evaluating referral outcomes for 500 pregnancies over the course of the 12 month study.

We generated descriptive statistics describing sociodemographic and practice for TBAs in both study arms. We also generated descriptive statistics for key baseline sociodemographic indicators and prior maternal and perinatal outcomes for patients cared for by the TBAs in both study arms. Only patients for whom written informed consent was obtained were included in this descriptive baseline analysis. Categorical data were reported as absolute numbers and percentages, and compared using the chi-square or Fisher’s exact test, as appropriate. Non-normally distributed continuous variables were reported as medians with interquartile ranges (IQR), and compared using the Wilcoxon-Mann-Whitney test.

For the primary outcome, in unadjusted intention-to-treat analysis, we compared the volume of monthly referrals for maternal and perinatal complications adjusted for monthly birth volume, using the Wilcoxon-Mann-Whitney test. Subsequently, to assess the effect of accessing the mHealth technology adjusting for seasonal variation and repeated-measures clustering by TBA, we also conducted an exploratory analysis using a generalized estimating equations (GEE) model (Poisson family distribution, log link function). For secondary outcomes, risk ratios (RR) with 95% confidence intervals (CI) were calculated. For all outcomes, a nominal *p* value of less than 0.05, without adjustment for multiple comparisons, was considered to indicate statistical significance. For patient-level outcomes, *p*-values were adjusted for clustering by TBA. All statistical analyses were conducted using Stata 13 (StataCorp, College Station, Texas).

## Results

### Participants

**Fig. 4:**
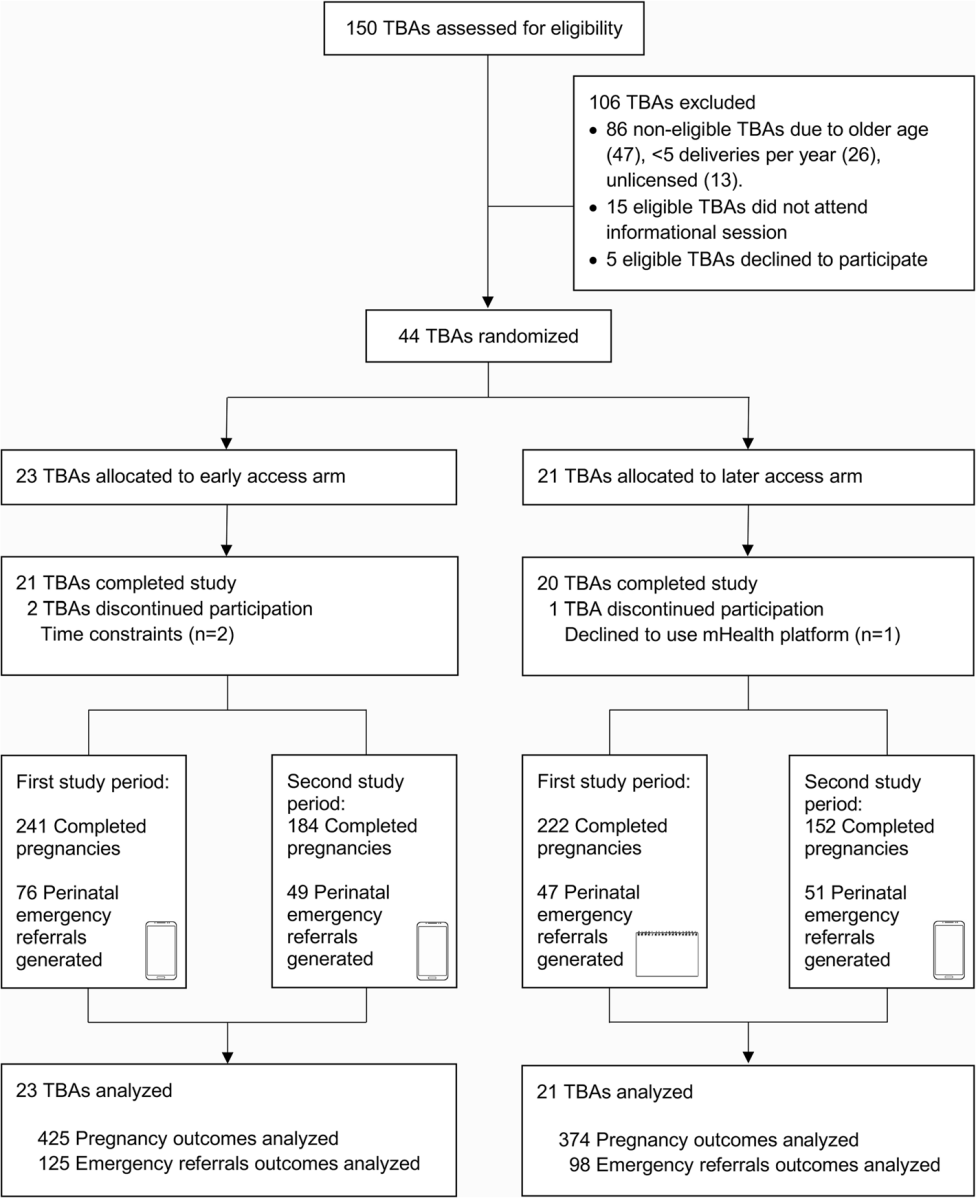
Study flow diagram. Enrollment, randomization, and follow-up

Forty-four TBAs consented participation in the study and underwent randomization (early-access arm: 23; later-access arm: 21; Fig. 4). Baseline demographic characteristics of participating TBAs in the two study arms were well balanced (Table 2). Eligible participant patients were recruited from April 2016 to March 2017. Throughout the study, TBAs reported 799 completed pregnancies (early-access arm: 241 completed pregnancies reported during the first study period, 184 completed pregnancies during the second study period; later-access arm: 222 and 152 respectively) (Fig. 4). Baseline demographic and clinical data were available for 335 patients in the early-access arm and 327 in the later-access arm. There were no statistically significant differences in baseline characteristics between patients in the two study arms (Table 3), except for proportion of women who experienced scarce economic resources as a barrier to obstetric care in the previous pregnancy (*p* = 0.009). Two TBAs in the early-access arm (9%) and one in the later-access arm (5%) discontinued their participation in the study but continued to report outcomes data, allowing for inclusion in the intention-to-treat analysis. Outcome and referral data was available for all completed pregnancies (Fig. 4).

**Table 2:** Baseline characteristics of study participants (traditional birth attendants)

Characteristic^a^	Early-Access Arm (*N* = 23)	Later-Access Arm (*N* = 21)
Age (years)	47 [40–55]	51 [43–55]
Education (years)	0 [0–2]	1 [0–4]
Spanish Literacy^b^	5 (22)	4 (19)
Monolingual Maya	13 (57)	9 (43)
Years in practice (years)	12 [10–20]	16 [10–24]
Distance from town (Km)	15 [5–21]	11 [7–18]
Travel time from town (min)	45 [15–60]	40 [15–60]

^a^Data are median [IQR], or n (%)

^b^Defined as able to read and write a simple sentence in Spanish

**Table 3:** Baseline demographic and clinical characteristics of patients treated by participating traditional birth attendants

Characteristic^a^	Early-Access Arm (*N* = 335)^b^	Later-Access Arm (*N* = 327)^b^	*p* value
Age (years)	26 [22–31]	26 [22–31]	0.59
Nulliparous	71 (21)	61 (19)	0.43
Number of pregnancies	3 [2–5]	3 [2–5]	0.73
Previous caesarian section^c^	27/263 (10)	33/263 (13)	0.41
Previous facility birth^c^	88/261 (34)	91/261 (35)	0.78
Previous perinatal complication^c^	84/265 (32)	83/263 (32)	0.97
Referral to hospital in last pregnancy^c^	60/262 (23)	59/262 (23)	0.92
Obstetric care barriers in last pregnancy	20/60 (33)	17/59 (29)	0.59
Lack of transportation	14 (70)	12 (71)	0.97
Scarce economic resources	8 (40)	14 (82)	0.009
Distance to hospital	4 (20)	5 (29)	0.51
Hospital mistreatment	5 (25)	2 (12)	0.31

^a^Data are median [IQR], or n (%)

^b^Where individual data was missing or not applicable for the entire sample, the denominator used to calculate each characteristic is given

^c^Calculated after excluding nulliparous individuals

### Outcomes

For primary outcomes, the rate of monthly emergency referrals over birth volume during the first study period (Fig. 3) was significantly higher (*p* = 0.03) for the early-access arm (median 33 referrals per 100 births, IQR 22 to 58; Table 4) in comparison to the later-access arm (median 20 referrals per 100 births, IQR 0 to 30; Table 4). During the second study period (Fig. 3) after the later-access arm transitioned to the mHealth platform, referral rates were not significantly different between the study arms (*p* = 0.58), with referral rate increasing as expected in the later-access arm after crossover into the mHealth intervention (Table 4). For secondary outcomes, the proportions of successful referrals were high (> 90%) for both study arms during both study periods (Table 4), without statistically significant differences. Non-successful referrals were due to refusal to referral (lack of permission to complete the referral from another family member (*n* = 5), patient’s fear to hospital due to cultural or language barriers (*n* = 4), or the patient not recognizing the complication as an emergency (n = 4)) or due to logistical difficulties during emergency communication or transportation (*n* = 3). To adjust for seasonal variation and repeated-measures clustering by midwife, we conducted an exploratory analysis using a generalized estimating equations (GEE) model; results from the GEE analysis similarly showed an increase in referral rates in the early-access arm, although this did not reach statistical significance (coefficient 1.24, 95% CI -0.93 – 3.40).

**Table 4:** Effect of intervention on emergency referral volume and successful referral completion by study arms

Outcome^1^	Early Access Arm (*N* = 23)	Later Access Arm (*N* = 21)	*p* value^2^
Adjusted monthly emergency referral rate (referrals/births) per 100 births			
First study period	33 [22–58]	20 [0–30]	0.03
Second study period	31 [10–42]	34 [5–50]	0.58
Referral success proportion (successful/referrals)			
First study period	69/76 (90.79)	44/47 (93.61)	0.74
Second study period	48/49 (97.95)	47/51 (92.16)	0.36

^1^Rates reported as median [IQR], and proportions as no./total no. (%)

^2^Wilcoxon-Mann-Whitney test for rates, Fisher’s exact test for proportions

Pregnancy outcomes and adverse events were monitored for both arms throughout the study. The study arms did not differ significantly with respect to mode of delivery (home, hospital, or caesarian delivery) (Table 5). There were no significant differences between study arms in the proportion of reported maternal and perinatal complications, with one exception: the rate of reported hypertensive disorders in pregnancy was higher among TBAs in the early-access arm than among TBAs in the later-access arm (3.53% vs 1.07%; *P* = 0.03). All complications reported in Table 5 are final diagnoses as determined at the time of emergency referral by the treating healthcare provider. Three maternal deaths due to preeclampsia or eclampsia were reported, with no difference in occurrence between the study arms (Table 5). Ten neonatal deaths were reported, with two thirds occurring in the first two days of life (6/10), and with no significant difference in occurrence between the study arms.

**Table 5:** Mode of delivery, reported complications, and adverse outcomes for completed pregnancies during the study

Outcome^1^	Early-Access Arm (*N* = 425)^2^	Later- Access Arm (*N* = 374)^2^	Risk Ratio (95% CI)	*p* value^3^
Mode of delivery				
Home delivery	345 (81.18)	318 (85.03)	0.95 (0.90–1.02)	0.35
Hospital delivery	42 (9.88)	29 (7.75)	1.27 (0.81–2.00)	0.43
Caesarian section	38 (8.94)	27 (7.22)	1.24 (0.77–1.99)	0.50
Emergency Referrals				
Maternal Complications				
• Labor progression abnormality	51 (12.00)	36 (9.63)	1.25 (0.83–1.87)	0.51
• Hypertensive disorders of pregnancy	15 (3.53)	4 (1.07)	3.3 (1.10–9.86)	0.03
• Hemorrhage	9 (2.12)	10 (2.67)	0.79 (0.33–1.93)	0.52
• Premature labor	5 (1.18)	6 (1.60)	0.73 (0.23–2.38)	0.64
• Fetal cardiac rate abnormality	4 (0.94)	4 (1.07)	0.88 (0.22–3.49)	0.84
Neonatal Complications				
• Suspected sepsis	4 (0.94)	5 (1.34)	0.70 (0.19–2.60)	0.62
• Respiratory compromise	5 (1.18)	2 (0.53)	2.2 (0.43–11.27)	0.46
• Preterm newborn	4 (0.94)	0	–	0.06
Death				
Maternal deaths	2 (0.47)	1 (0.27)	1.76 (0.16–19.33)	0.65
Neonatal deaths	7 (1.65)	3 (0.80)	2.05 (0.53–7.88)	0.23

^1^Outcomes reported as no. (%)

^2^*N* = reported births. All births were singleton

^3^*p* values are adjusted for clustering by TBA using logistic regression

## Discussion

This pragmatic, randomized-controlled feasibility trial showed that the introduction of a mHealth decision support technology into the routine community-based practice of a group of TBAs in rural Guatemala resulted in a significant increase in the rate of emergency referrals to facility-care for maternal and perinatal complications. We designed this study with the goal of conducting foundational implementation research on the utility of smart phone technology to improve health decision-making and linkages to higher-level obstetrical care by TBAs, the de facto front line health workers for rural, indigenous women in much of Guatemala. Using a pragmatic, randomized controlled design, we demonstrated that TBAs who received early access to a smartphone-based decision support technology, integrated into their routine community-based practice, had increased monthly referral rates compared to those who provided usual care, even when given manual access to referral processes via training and a laminate reminder card. Furthermore, when TBAs in the usual care arm subsequently received access to the technology, their referral rates also increased similarly to the early-access arm. Interestingly, referral success rates were very high for both arms throughout the study. This suggests that, at least within the MHA institution context which provides meaningful wrap-around support to coordinate emergency referral, the most important impact of the mHealth intervention is on increasing detection of complications.

Rates of most maternal and neonatal complications were similar in both study arms. Although one might expect that increased detection of early warning signs and referral to higher-level care might decrease complication rates, this was an exploratory feasibility study. Baseline complication rates were unknown for the study population, and the study was not powered to detect a difference in the rate of any complication. However, we did find an increase in the proportion of hypertensive disorders detected in the early-access arm. This finding is remarkable, because use of the mHealth technology here included an automated oscillometric blood pressure monitoring (Fig. 1). This suggests that the mHealth technology improved detection of hypertensive disorders of pregnancy, which are a leading cause of maternal and perinatal deaths [19–21]. In fact, in an analysis of data from the international Global Network for Women’s and Children’s Health Research Maternal and Neonatal Health Registry—which includes data from the same region of Guatemala where this study occurred—hypertensive disorders were the strongest predictor of maternal death [22]. In-home blood pressure monitoring is not routinely available or utilized by TBAs in rural Guatemala, and integrating this technique into their skillset as our intervention does here has the potential to directly impact the high rates of maternal and perinatal mortality [23, 24].

Our study has several strengths. To our knowledge, it is the first attempt to quantify baseline TBA-initiated referral rates for maternal and neonatal complications in the region. These data will be important both for policy and public health initiatives as well as for planning large-scale clinical trials. Secondly, pragmatic incorporation of the mHealth intervention into an existing primary care collaboration linking TBAs to the formal healthcare system allowed for more direct evaluation of the hypothesis that mHealth decision support could increase referral rates. The overall high proportion of successful referrals before and after access to the mHealth technology suggests that the quality of the wrap-around support environment for TBAs was similar for both study arms while using or not the mHealth platform and that, therefore, the increase in rate of referral was due to use of the platform. Finally, TBAs attrition was low and the use of the mHealth platform was high, despite the low literacy and lack of previous exposure to technology. This suggests that the research team’s use of agile design with early end-user feedback [16, 17] helped produce a functional system which integrated easily into the usual daily practice of participating TBAs. Potential limitations of our study should be considered. This was a pragmatic feasibility trial designed to explore the use of mHealth to improve referral volume. Given this context and the fact that the intervention involved introduction of sophisticated technology, it was not possible to blind study staff and TBAs to study arm allocation. Additionally, the study was not powered to detect differences in clinical outcomes. Finally, due to the unidirectional cross-over study design, there was no complete control arm without any access to the intervention. Furthermore, TBAs in the study self-reported pregnancy and referrals outcomes, which may have led to underreporting of patients and pregnancies with certain characteristics or unfavorable outcomes. Effects of possible confounders, particularly temporality, on secondary patient-level endpoints were not addressed, given the small overall sample size and small number of observed complications. Our study took place in rural Guatemala in collaboration with indigenous Maya TBAs and patients and, therefore, our technology intervention and findings may not be generalizable to different settings or populations. Finally, in exploratory analysis, referral rates (although encouraging) were not significantly different when adjusted for repeated-measures clustering. This may be a simple statistical power issue which will be addressed in a future study powered to detect these adjusted differences. However, it also illustrates the need, in future work, to better define and address variation in TBAs’ practice parameters and ability to optimize use of the mHealth technology.

This study is, to our knowledge, the first report of supportive mHealth technology for low-literate TBAs with little previous exposure to technology in a challenging rural healthcare delivery context, as well as the first report of referral rates by TBAs in indigenous populations, and it adds to the evidence of community-based intervention packages for improving maternal and perinatal outcomes [25, 26] and of mHealth interventions integrated within existing healthcare systems as potential solutions for addressing maternal health in LMICs [13, 27–29]. For example, in a study conducted in rural Pakistan by Jokhio et al. [30], training TBAs to recognize maternal and perinatal complications, alongside efforts made to better integrate them into the formal health system, resulted in increased referral rates to facility-care for emergency obstetrical care. Similarly, a recent study in rural Guatemala connecting community health workers – with higher levels of clinical training and literacy than the TBAs in our study – to medical specialists via cell-phones [31], showed a promising trend toward reductions in maternal mortality.

In future research, we plan to validate incorporation of mHealth decision support into TBA workflows in a larger scale study adequately powered to detect differences in important maternal and perinatal clinical outcomes and to include a cost analysis of our intervention. By providing estimates of baseline referral and complication rates, as well as observations on variations in individual TBA practice, this pilot feasibility study will assist in planning that study. We are also in the process of collecting and analyzing qualitative feedback from TBAs, patients, and staff at health facilities on the systems’ impact, in terms of improving the quality of healthcare for women and the integrations of TBAs into the healthcare system. Our overall goal remains improving linkages of TBAs in rural Guatemala to the formal healthcare system and respectful maternity care for indigenous mothers [32].

## Conclusion

Our trial showed an increase in the rate of referrals to higher-level obstetrical care among TBAs utilizing a mHealth decision support platform. Our results show that the introduction of mHealth technologies for TBAs is feasible and may improve referral to facility-care within a challenging healthcare delivery context such as rural Guatemala. As an early-stage pragmatic trial, our findings are subject to many limitations. Therefore, further research needs to be conducted to explore the effects of mHealth systems on perinatal and maternal morbidity and mortality and to rigorously examine the impact of temporal trends and individual TBA practice variation on obstetrical referral. Nevertheless, despite these limitations, our promising results help make a strong case for continued investments in TBA training and technologies to improve linkages of TBAs to the formal healthcare system.

## Abbreviations

LMIC: Low- and middle-income country
MHA: Maya Health Alliance
mHealth: Mobile Health
TBA: Traditional birth attendant

## References

[1] United Nations. Sustainable Development Goals. http://www.un.org/sustainabledevelopment/health/ (2015). Accessed 10 Oct 2017.

[2] World Health Organization (2015). Maternal mortality. Fact sheet #348.

[3] World Health Organization (2016). Maternal and perinatal health.

[4] The World Bank. Indicator: Maternal mortality ratio.http://data.worldbank.org/indicator/SH.STA.MMRT (2015). Accessed 10 Oct 2017.

[5] The World Bank. Indicator: Mortality rate, under-5 (per 1,000 live births). http://data.worldbank.org/indicator/SH.DYN.MORT (2016). Accessed 11 Oct 2017.

[6] Stollak I, Valdez M, Rivas K, Perry H (2016). Casas Maternas in the rural highlands of Guatemala: a mixed-methods case study of the introduction and utilization of birthing facilities by an indigenous population. Glob Health Sci Pract.

[7] Ministerio de Salud Pública y Asistencia Social (2015). VI Encuesta Nacional de Salud Materno Infantil.

[8] Walsh LV (2006). Beliefs and rituals in traditional birth attendant practice in Guatemala. J Transcult Nurs.

[9] Goldman N, Glei DA (2003). Evaluation of midwifery care: results from a survey in rural Guatemala. Soc Sci Med.

[10] Chary A, Díaz AK, Henderson B, Rohloff P (2013). The changing role of indigenous lay midwives in Guatemala: new frameworks for analysis. Midwifery.

[11] Garces A, Mcclure EM, Hambidge KM, Krebs NF, Figueroa L, Aguilar ML (2015). Trends in perinatal deaths from 2010 to 2013 in the Guatemalan western highlands. Reprod Health.

[12] Homer CS, Friberg IK, Dias MA, ten Hoope-Bender P, Sandall J, Speciale AM (2014). The projected effect of scaling up midwifery. Lancet.

[13] Colaci D, Chaudhri S, Vasan A (2016). mHealth interventions in low-income countries to address maternal health: a systematic review. Ann Glob Health.

[14] Munro ML, Lori JR, Boyd CJ, Andreatta P (2014). Knowledge and skill retention of a mobile phone data collection protocol in rural Liberia. J Midwifery Womens Health.

[15] Lori JR, Munro ML, Boyd CJ, Andreatta P (2012). Cell phones to collect pregnancy data from remote areas in Liberia. J Nurs Scholarsh.

[16] Stroux L, Martinez B, Coyote Ixen E, King N, Hall-Clifford R, Rohloff P (2016). An mHealth monitoring system for traditional birth attendant-led antenatal risk assessment in rural Guatemala. J Med Eng Technol..

[17] Martinez B, Hall-Clifford R, Coyote E, Stroux L, Valderrama CE, Aaron C (2017). Agile development of a smartphone app for perinatal monitoring in a resource-constrained setting. J Health Inform Dev Ctries.

[18] Leon AC, Davis LL, Kraemer HC (2011). The role and interpretation of pilot studies in clinical research. J Psychiatr Res.

[19] Say L, Chou D, Gemmill A, Tunçalp Ö, Moller AB, Daniels J (2014). Global causes of maternal death: a WHO systematic analysis. Lancet Glob Health.

[20] Kassebaum NJ, Bertozzi-Villa A, Coggeshall MS, Shackelford KA, Steiner C, Heuton KR (2014). Global, regional, and national levels and causes of maternal mortality during 1990–2013: a systematic analysis for the global burden of disease study 2013. Lancet.

[21] World Health Organization. WHO recommendations for prevention and treatment of pre-eclampsia and eclampsia. Geneva: World Health Organization; 2011. 23741776

[22] Bauserman M, Lokangaka A, Thorsten V, Tshefu A, Goudar SS, Esamai F (2015). Risk factors for maternal death and trends in maternal mortality in low-and middle-income countries: a prospective longitudinal cohort analysis. Reprod Health.

[23] Garces A, McClure EM, Chomba E, Patel A, Pasha O, Tshefu A (2012). Home birth attendants in low income countries: who are they and what do they do?. BMC Pregnancy Childbirth.

[24] Milne F, Redman C, Walker J, Baker P, Bradley J, Cooper C (2005). The pre-eclampsia community guideline (PRECOG): how to screen for and detect onset of pre-eclampsia in the community. BMJ.

[25] Lassi ZS, Bhutta ZA. Community-based intervention packages for reducing maternal and neonatal morbidity and mortality and improving neonatal outcomes. Cochrane Database Syst Rev 2015; Issue 3. Art. No.: CD007754. https://doi.org/10.1002/14651858.CD007754.pub3. 10.1002/14651858.CD007754.pub3 PMC8498021 25803792

[26] ten Hoope-Bender P, de Bernis L, Campbell J, Downe S, Fauveau V, Fogstad H (2014). Improvement of maternal and newborn health through midwifery. Lancet.

[27] Tamrat T, Kachnowski S (2012). Special delivery: an analysis of mHealth in maternal and newborn health programs and their outcomes around the world. Matern Child Health J.

[28] Mechael P, Batavia H, Kaonga N, Searle S, Kwan A, Goldberger A, et al. Barriers and gaps affecting mHealth in low and middle income countries: Policy white paper. 2010. http://www.globalproblems-globalsolutions-files.org/pdfs/mHealth_Barriers_White_Paper.pdf. Accessed 18 Jan 2018.

[29] Clifford GD (2016). E-health in low to middle income countries. J Med Eng Technol.

[30] Jokhio AH, Winter HR, Cheng KK (2005). An intervention involving traditional birth attendants and perinatal and maternal mortality in Pakistan. N Engl J Med.

[31] Martínez-Fernández A, Lobos-Medina I, Díaz-Molina CA, Chen-Cruz MF, Prieto-Egido I (2015). TulaSalud: an m-health system for maternal and infant mortality reduction in Guatemala. J Telemed Telecare.

[32] Austad K, Chary A, Martinez B, Juarez M, Juarez Martin Y, Coyote Ixen E (2017). Obstetric care navigation: a new approach to promote respectful maternity care and overcome barriers to safe motherhood. Reprod Health.

[33] FARMAMUNDI, ASECSA, MSPAS, KOICA, UNFPA y OSAR. Más que una sanadora, Manual práctico de comadronas tradicionales para una maternidad sana. Guatemala. 2014.

